# Development of the human pancreas and its exocrine function

**DOI:** 10.3389/fped.2022.909648

**Published:** 2022-09-29

**Authors:** Vijay Mehta, Puanani E. Hopson, Yamen Smadi, Samit B. Patel, Karoly Horvath, Devendra I. Mehta

**Affiliations:** ^1^Center for Digestive Health and Nutrition, Arnold Palmer Hospital for Children, Orlando, FL, United States; ^2^Department of Children Center, Pediatric and Adolescent Medicine, Gastroenterology and Hepatology, Mayo Clinic, Rochester, MN, United States; ^3^Pediatric Gastroenterology and Nutrition of Tampa Bay, Tampa Bay, FL, United States

**Keywords:** pancreas, ontogeny, genes, development, prenatal and postnatal of enzyme secretion

## Abstract

The pancreas has both endocrine and exocrine function and plays an important role in digestion and glucose control. Understanding the development of the pancreas, grossly and microscopically, and the genetic factors regulating it provides further insight into clinical problems that arise when these processes fail. Animal models of development are known to have inherent issues when understanding human development. Therefore, in this review, we focus on human studies that have reported gross and microscopic development including acinar-, ductal-, and endocrine cells and the neural network. We review the genes and transcription factors involved in organ formation using data from animal models to bridge current understanding where necessary. We describe the development of exocrine function in the fetus and postnatally. A deeper review of the genes involved in pancreatic formation allows us to describe the development of the different groups (proteases, lipids, and amylase) of enzymes during fetal life and postnatally and describe the genetic defects. We discuss the constellation of gross anatomical, as well as microscopic defects that with genetic mutations lead to pancreatic insufficiency and disease states.

## Introduction

The pancreas has both endocrine and exocrine functions. The endocrine system consists of multiple peptide hormones that function to regulate blood glucose but also influence exocrine functions (e.g., somatostatin). Its exocrine function involves the secretion of enzymes, bicarbonate, and water to aid in the digestion of nutrients.

There are many unanswered questions related to embryonic pancreatic development. Due to ethical issues, research in human development of the pancreas has been limited and predominantly conducted *via* animal studies.

In this review, we describe the current understanding of the development of the human pancreas, including gross and microscopic anatomy with a focus on exocrine function. In addition, we will describe disease states as the consequence of abnormalities in pancreas development and the genetic mutations behind them. We will utilize animal models to highlight the possible development of the human pancreas and its implications in disease states.

## Intrauterine pancreas development

The foregut endoderm gives rise to the dorsal and ventral pancreatic buds between days 26 and 31 of embryonic development.

**Figure 1:**
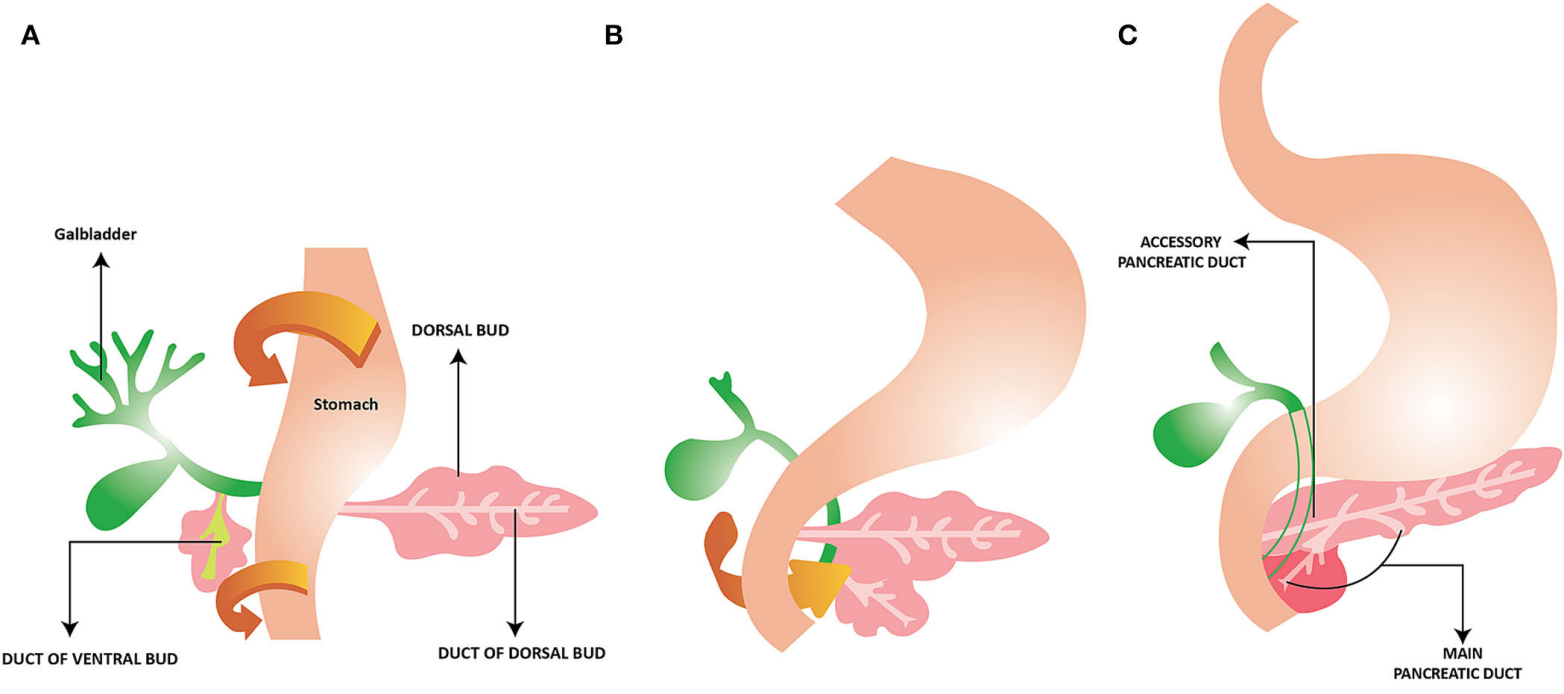
Development of the Pancreas. **(A)** Rotation of the ventral bud with the stomach and duodenum to the right. **(B)** The ventral and dorsal bud fuse together. **(C)** The main pancreatic duct exiting via the ventral bud to the duodenum. Ventral bud gives rise to uncinate process and part of the head of the pancreas. The dorsal bud gives rise to the remainder of the pancreas. Reprinted from “Pancreatic Duct Variations and the Risk of Post-Endoscopic Retrograde Cholangiopancreatography Pancreatitis” Ojo A S. Cureus, 12 (9): e10445. 2020 by Cureus.

Initially, there are two ventral pancreatic buds but the left side regresses. Figure 1 depicts the rotation of the stomach and duodenum starting at week 5 (Figure 1A) which results in the fusion of the two buds with the ventral bud lying posterior by week 6 (Figure 1B). The majority of the organ is derived from the dorsal bud while the ventral bud will give rise to the uncinate process and part of the head of the pancreas (Figure 1C) (1–3).

### Pancreatic ducts

The main pain of the pancreatic duct (duct of Wirsung) is formed from the pancreatic duct of the ventral bud and the distal part of the duct of the dorsal bud. The proximal part of the dorsal bud contains an accessory pancreatic duct that opens into the minor duodenal papilla (4, 5). Initially, the ducts of the dorsal and ventral pancreas fuse, followed by partial regression of the dorsal pancreatic duct proximal to the duodenum to form Santorini's canal (6). In some, this will open into the minor papilla, while in others, it will be non-draining or connect with the main duct (6–8). Lack of fusion of the ventral and pancreatic ducts results in pancreatic divisum (9). By the 8th week, the bile tree and main pancreatic duct are joined together at the duodenum (10).

Three variants have been noted in the location of biliary and pancreatic ducts. The most common is when the pancreatic and bile duct joins at variable distances from the duodenum. The second variant includes joining at the duodenal wall and the third variant is when the pancreatic duct and biliary duct open at separate locations in the duodenum. While no particular pathology has been associated, there may be potential for pancreatitis in some individuals with the second and third variants (7, 11). These variations are important during the time of surgery or ERCP (11, 12).

### Cellular matrix

Based on animal models the formation of cells that make up the pancreas starts as the epithelium of the buds begins to fold. It is followed by phases of branching, proliferating, and differentiating. The result is grape-like clusters, in which islet cells form clusters to the periphery of acinar and ductal cells (5).

The pancreas is made predominantly of acinar and duct cells, while the islet cells make up 1 to 2% of the pancreas. By the 9th week, the pancreas exists as tubules and clusters of undifferentiated epithelial cells. The tubules continue to grow, followed by lobule formation by the 14th week. Acinar cells with zymogen granules are noted between the 12th and 15th week, and in significant numbers by the 20th week (13). During this phase, the endoplasmic reticulum and Golgi apparatus undergo significant maturation (6). This is followed by progressive growth with a lumen in the center and lobules to the periphery with numerous acini (6).

Pancreatic stellate cells secrete extracellular matrix proteins and seem to have a significant function in the first trimester of human pancreatic development by enhancing differentiation to exocrine cell lineages (14).

### Neuronal network

Comparable with the enteric nervous system, an intra-pancreatic nervous system develops to enable a degree of independence of the pancreas from the central nervous system and the gut. The pancreatic ganglia are the nervous integration centers of the pancreatic exocrine and endocrine secretion. Vagal preganglionic, sympathetic postganglionic, sensory, and enteric fibers innervate the fully developed pancreas. Postganglionic nerve fibers surround almost every acinus, forming a periacinar plexus containing cholinergic, noradrenergic, peptidergic, and nitrergic fibers, which terminate at the acinar cells. The autonomic nervous system of the pancreas interacts with ganglionic structures that are randomly scattered throughout the pancreatic parenchyma and represent the intrinsic neural component of the pancreatic nerve supply (15). Neurons and nerve fibers form complexes with endocrine cells, and epithelial cells located in ducts early in the second trimester and include pain fibers from parasympathetic nerves (16). Interestingly, innervation is found most densely at the head of the pancreas and decreases toward the tail (17). The sympathetic system seems to develop during this early fetal period and may have a role in endocrine pancreas development (18). Understanding sympathetic innervation of the exocrine pancreas is in its infancy. Studies seem to demonstrate sympathetic inhibition of blood flow, resulting in decreased exocrine secretion, and therefore indirect effect (19). On the other hand, parasympathetic innervation has a large role in exocrine function, mediated *via* vagal nerve activity, especially in the cephalic phase (20). Gastric, intestinal, and absorbed nutrient phases also appear to have both direct and indirect (*via* vagal nerve) mechanisms of pancreatic enzyme secretion (21). See *Neural Control of the Pancreas | Pancreapedia* for more information.

## Development of exocrine function

The major adult human digestive exocrine pancreatic enzymes include amylase, pancreatic triglyceride lipase (PTL), colipase, trypsinogen, chymotrypsinogen, carboxypeptidase A1 and A2, and elastase. We will discuss the enzymes found in the intrauterine period followed by postnatal development and the maturation process to adult levels of the enzymes.

### Intrauterine period

Most of the human pancreas exocrine development is derived from morphological studies. The exocrine tissue comprises acinar cells that secrete digestive enzymes and a duct system that deliver them to the small intestine.

Pancreatic secretory trypsin inhibitor (*PSTI*) or serine protease inhibitor kazal type 1 (*SPINK1*) was first noted by immunohistochemistry at the 8th gestational week (22).

### Proteases

Adult human proteases include trypsinogen, chymotrypsinogen, elastase 1, and carboxypeptidase A1 and A2.

Many enzymes, including trypsinogen, chymotrypsinogen, and elastase-1, appear between 14th and 16th weeks of gestation (22–24).

The trypsinogen and chymotrypsinogen appear to be present from 16th weeks gestation and increase until birth (23). Activation of trypsinogen to trypsin requires enterokinase, while chymotrypsinogen requires trypsin for conversion to chymotrypsin (25, 26). Chymotrypsin was present in the 23-week premature infants with levels similar to term infants (27). While initially the development of enterokinase was thought to be around 26th week gestation, the levels of chymotrypsin found by Kolacek et al. suggest this may be slightly earlier (27, 28).

Neonates from 32 weeks gestation to term appear to have 90% to 100% of trypsin levels of children 2 years of age. On the other hand, levels of chymotrypsin is 50-60% and while carboxypeptidase B is 10-25% the level of 2-year-old children, thus showing the development of protease levels over time (Table 1) (29).

**Table 1:** Ontogeny of common pancreatic enzymes in humans.

**Pancreatic enzyme**	**Prenatal development (Gestational week)**	**Postnatal activities**	**Adult level**	**References**
Amylase	39 weeks	< 1% of adult values or absent levels until 6 weeks	6 months to 2 years	(22, 29–32)
Pancreatic triglyceride lipase	13–21 weeks	5–10% of adult values	2 years	(29, 33–35)
Trypsin *	14–16 weeks	90–100% of adult values	< 1month	(22, 23, 29, 30)
Chymotrypsin*	14–16 weeks	50–60% of adult values	2 years	(22, 23, 29, 30, 36)
Elastase	14–16 weeks	25% of adult values	2 weeks	(22, 37)

*Initially detected as trypsinogen and chymotrypsinogen.

#### Lipases

Pancreatic triglyceride lipase (PTL) is the predominant lipase in human adults. However, other enzymes involved in fat breakdown include colipase and phospholipase A and B.

Lipases such as carboxyl ester lipase and pancreatic lipase-related proteins 1 and 2 (*PLRP*) were present from 14 to 16 weeks gestation (22–24), though *PLRP1* has no known activity (38). The mRNA encoding *PLRP1* and *PLRP2* was present by the 16th week in the human fetal pancreas. In contrast, the mRNA encoding PTL is limited in the fetal pancreas (29), and likely does not start increasing until 41 weeks gestation (24).

#### Amylase

Pancreatic amylase (encoded by *AMY2*) is present in adult humans but has not been detected in the fetus, and in fact is at low levels in humans even until 3 months of age (22, 39, 40).

### Postnatal maturation of the exocrine function

Digestion of lipid, protein, and carbohydrates in infants relies on the state of the maturation of exocrine pancreatic function. However, diet composition can also affect enzyme production. Infants do not respond to exogenous cholecystokinin (CCK) or secretin well, but the exact maturation time of response of the exocrine pancreas to secretagogues is not well-defined (41, 42). Understanding normal age-based values of pancreatic enzyme activity in duodenal fluid with pancreatic function testing would allow a better understanding of the ontogeny of pancreatic exocrine tissue.

Interestingly, a diet in the form of starch and protein augments the production of α-amylase and trypsin, respectively, but fat does not stimulate the increased lipase levels in infants (36). However, a high protein and low-fat diet stimulates both trypsin and PTL activity (36). After 12 months of age, PTL does appear to be stimulated over baseline activity by meals (29). It may therefore be possible that CCK and secretin more effectively stimulate exocrine function closer to 12 months of age.

Initially, infants have “physiologic” steatorrhea in the first 3 to 6 months postnatally. As previously noted, lipase is at very low levels in the neonatal period. Based on rodent models, *PLRP2* may have a role in triglyceride digestion in newborns (38). Along with *PLRP2*, the presence of colipase has been noted, which based on animal models appears to increase the activity of *PLRP2* (43). Overall, PTL output or the coefficient of fat absorption is 5 to 10% of the adult values (29, 33, 44). In a study by Track et al., the PTL levels at 3 weeks were significantly higher than at 3 days of life and approached that of adult levels (34). However, in the study by Lebenthal et al., the PTL levels were low at birth and 1 month, with a substantial rise by 2 years (Table 1) (29, 30).

Amylase in the acinar cells was not detected until 39 weeks gestational age, and the functional amount of amylase does not arise until the 6th week postnatally (22, 29). In premature infants, amylase activity does not increase after a meal, compared to trypsin, and it is presumed that pancreatic amylase needs are met in the form of salivary amylase and amylase in breastmilk (29, 45). Indeed, it is thought that amylase remains at low levels until 6 months, although isolated amylase deficiency was noted to be frequent till 2 years of age (Table 1) (31, 32). Interestingly, in one cohort, isolated amylase deficiency had been noted beyond 2 years of age with a prevalence of up to 3.5% (32).

Elastase on the other hand is found at low levels at birth in meconium but reaches levels at the lower end of normal within 3 to 4 days in term infants and 2 weeks in preterm infants (Table 1) (37).

The immaturity of exocrine pancreas function is a notable factor in infant's vulnerability to metabolic and nutritional stress (33). Thus, additional non-pancreatic sources of digestion are present. Breast milk can be a source of amylase and bile salt-stimulated lipase. Additionally, brush-border glucoamylase is present in newborns with similar concentrations to that of adults, which may help in the digestion of complex carbohydrates.

## Genes involved in the pancreas development

The process for the development of the pancreas and the fetus is based on specific coordination between genetics and the local environment. Much of our understanding of genes and molecular signaling comes from animal studies with the assumption that human development is similar. Table 2 summarizes genes and roles in pancreas development. We will discuss some of the genes that are involved in pancreas development and more specifically acinar and ductal cells. For further details, please visit *Development of the Pancreas | Pancreapedia* and work by Jennings et al. (47).

**Table 2:** Transcription factors and cell signaling involved in human pancreas development.

**GENE**	**ROLE**	**References**
Wnt/ β-catenin	Inhibition in endoderm allows for pancreas and liver development. Later activation is required for cellular growth, specifically acinar cells	(46)
PDX1	Important in early pancreas formation, is found in multipotent progenitor cells as well as ductal and endocrine cells. Mutations have been associated with pancreatic agenesis as well as MODY	(47–51)
Sonic Hedgehog (SHH)	Signaling from notochord decreases activity of Shh, allowing *PDX1* expression	(47, 52)
PTF1A	Has a role along with *PDX1* in pancreas development and formation, as well as a crucial role in acinar cell development	(53–55)
GATA4	Transcription factor involved in early dorsal and ventral pancreatic bud formation. Later is found in progenitor and peripheral Tip cells, and finally acinar cells Association with neonatal diabetes and possible exocrine insufficiency	(56, 57)
NEUROG3	Appears to be present by 8 weeks, and disappears by 35 weeks, and is important in endocrine islet cell formation	(58, 59)
SOX9	Transcription factor in determining cell types and is uniformly present in ductal cells. Additionally has a role in ventral and dorsal bud formation. Mutation results in multiple systemic abnormalities, including pancreatic hypoplasia	(56, 60)
HNF1B	Involved in pancreas formation and ductal cell lineage early on in embryogenesis. Mutations have been associated with pancreas agenesis and MODY	(61–64)

After the initial formation of pancreatic tissue, activation of *Wnt/*β*-catenin* is required for pancreatic tissue growth, particularly in acinar cells (46). Inhibition of another protein, sonic hedgehog (*SHH*), likely due to the proximity of the notochord, allowed for future expression of pancreas-duodenum homeobox 1 (*PDX1*), also known as insulin promoter factor 1 (IPF1) (47, 52). *PDX1* has been found as early as pancreatic bud formation and continues to be present in all epithelial cells in the embryonic period, and remains in the nuclei of non-endocrine epithelial cells along with the nuclei and cytoplasm of islet cells into adulthood (48).

Acinar cells begin initially as several carboxypeptidase A1 pyramidal cells bud from the pancreatic epithelium, likely representing the proacinar population. Additionally, *GATA4*, basic helix-loop-helix transcription factor (*MIST1*), and pancreatic secretory trypsin inhibitor are also expressed (1). However, the seminal work by Jennings et al. (56) suggests forkhead box protein A2 (*FOXA2*), and SRY-related homeobox 9 (*SOX9)* are other predominant transcription factors that define cell types. In particular, *SOX9* and *FOXA2* are present in ductal cells, while *GATA4* is present in acinar cells from the second trimester onward (56). *SOX9* has been noted in dorsal and ventral buds from day 32 with higher levels found in later embryonic stages days 44 to 52 (60). Other cellular markers of ductal cells include cytokeratin 19 (CK19) and CD133. CD133 is found in fetal duct-like cells, while CK19 is widely expressed in fetal pancreatic epithelial cells and continues as a ductal cell marker in adults (48, 65).

Notch signaling interacts with recombination signal binding protein for immunoglobulin kappa J region (*RBPJ*) to downregulate neurogenin-3 (*NEUROG3*) expression *via* hairy and enhancer of split-1 (*HES1*) (66). NEUROG3 is involved in endocrine commitment. *HES1* along with pancreas-associated transcription factor 1a (*PTF1a*) promotes acinar development (5).

As previously noted, *PTF1a* is important in the development of acinar cells. This is a class B basic helix-loop-helix (bHLH) transcription factor, which appears to be conserved across species. In animal models, the development of pancreatic buds includes *PTF1a* along with *PDX1* expression (67). However, further development of the pancreas includes an enhancer that binds to the active form of *PTF1a* and *RBPJ* to regulate *PTF1a* expression in acinar cells (68). Over time RBPJ is replaced with RBJPL which in complex with PTF1A and other class A bHLH forms PTF1-L and drives downstream regulators of digestive enzymes (53, 69, 70).

## Structural abnormalities in pancreas development

Detailed description of developmental abnormalities that are associated with pancreatitis are outside the scope of this review. For completeness we describe them in brief.

### Pancreatic divisum

Pancreatic divisum is the most common abnormality related to ductal fusion and drainage. It occurs in up to 10% of individuals (9). The lack of pancreas fusion results in drainage of the dorsal part of the pancreas *via* the minor papilla, while the ventral portion drains the head of the pancreas, uncinate process, and biliary tree into the major duct (71). It remains unclear if this anomaly increases the risk of recurrent pancreatitis and chronic pancreatitis.

### Annular pancreas

The annular pancreas is a rare cause of pancreatitis and duodenal obstruction, typically with non-bilious emesis (72). It results in a thin band of pancreatic tissue surrounding the second part of the duodenum. It is hypothesized that the two ventral buds remain and fuse with the dorsal bud to create the ring (4). This may be the result of abnormal hedgehog signaling (5). Within the annular pancreas, there are six further classifications of type of annular pancreas depending on ductal drainage, including the main duct, minor duct, and common bile duct (73, 74).

### Pancreatobiliary malformation

Anomalous pancreaticobiliary junction results from the joining of bile and pancreatic ducts outside the duodenal wall. It is believed that dysplasia and misarrangement of the bile and ventral pancreatic duct around the time of ventral pancreas formation results in pancreaticobiliary malformation (PBM) (75, 76). Typically, the end of the common bile duct is surrounded by the papillary sphincter, regulating bile flow while preventing reflux of pancreatic juices (77). However, with PBM, the sphincter is more distal (more than 15 mm) to the junction of the bile and pancreatic duct. As a result, bile and pancreatic juices may reflux into the other respective ducts, resulting in inflammation, bile duct dilation, pancreatitis, bile duct, and gallbladder cancer (78, 79).

It is further divided into three types, bile duct, pancreatic duct, and complex type, based on each joining at an acute angle. Reflux into the biliary system is thought to result in a higher risk for congenital choledochal cyst, and, in particular, type Ia, Ic, and IV-A are associated with PBM (80). The biliary system is particularly susceptible as the pressure in the pancreatic duct is generally higher (81).

### Ansa pancreatica

The **ansa pancreatica** is a rare type of anatomical variation of the pancreatic duct. It is a communication between the main pancreatic duct (of Wirsung) and the accessory pancreatic duct (of Santorini). Ansa pancreatica has been considered a predisposing factor in patients with idiopathic acute pancreatitis (82).

## Genetic diseases with compromised exocrine function

The most common genetic disorder resulting in exocrine pancreatic insufficiency include Cystic fibrosis (>90%), followed by Schwachman-diamond, Johanson-Blizzard, Pearson's bone marrow, pancreatic agenesis/hypoplasia, isolated enzyme deficiencies, and genetic or metabolic causes of pancreatitis (83). These will be discussed here in brief for completeness.

### Cystic fibrosis

Cystic fibrosis transmembrane conductance regulator (*CFTR*) is found in ductal epithelial cells and is involved in HCO^3^ - and Cl^−^ transport between the membranes along with water (84). Impaired movement of the ions, and fluid, results in increased viscosity and obstruction of the lumen (85). It is hypothesized that pancreatic insults begin in utero and progress after birth to affect all ducts resulting in pancreatic insufficiency (86). Pancreatic insufficiency occurs in 85% of this population, with a high prevalence among those with homozygous mutations for Δ*508* (83).

### Schwachman-diamond syndrome

Schwachman-Diamond syndrome is an autosomal recessive disorder with bone marrow involvement, skeletal abnormalities, and pancreatic insufficiency with variable penetrance (87). Mutations predominantly on chromosome 7 seem to be responsible, but with a spectrum of homozygous and compound heterozygous mutations (88, 89). It has been identified in 90% of individuals with this syndrome and the protein produced by the gene appears to affect ribosome function and may reduce protein production (90). Biopsies of the pancreas have shown that the duct and islet cell functions are preserved; however, acini are replaced by adipose tissue (91). Interestingly, over time, it seems that patients regain some function, and 40% to 60% have sufficient exocrine function (92).

### Johanson-Blizzard syndrome

Johanson-Blizzard syndrome is an autosomal recessive disorder with a defect in the *UBR1* gene on Chromosome 15 (15q14–21.1) (93). E3 Ubiquitin ligase UBR1 is involved in the breakdown of intracellular pathways, thus mutations in this affect appropriate protein recycling (94). The typical clinical features are exocrine pancreatic insufficiency, hypoplasia/aplasia of the alae nasi, congenital scalp defects, and growth retardation (95, 96). Zenker et al. reported that individuals with this syndrome did not express *UBR1* and had intrauterine-onset destructive pancreatitis with secondary replacement of acinar cells with adipose tissue resulting in pancreatic insufficiency (93, 97, 98).

### Pearson syndrome

Pearson syndrome is characterized by the bone marrow with vacuolization of erythroid and myeloid precursors, and sideroblasts, along with pancreatic insufficiency and it was initially reported by Pearson (99). It was later confirmed that this syndrome is the result of mitochondrial DNA deletion, affecting protein-coding and tRNA genes and therefore mitochondrial structure and function (100, 101). However, the prevalence of pancreatic insufficiency varies, likely due to variation in mitochondrial DNA deletions (100, 102–104).

### Pancreatic agenesis

Agenesis of the dorsal pancreas can have non-specific findings, including abdominal pain, and often requires imaging findings such as CT to show a lack of pancreatic tissue (105). Several transcription factors have been implicated in pancreas malformation and agenesis, these include *PDX1*, Hepatocyte nuclear factor (*HNF1B*), *PTF1A*, and *SOX9*. A case report described a homozygous point deletion mutation in *PDX1* resulting in pancreatic agenesis with resulting exocrine and endocrine insufficiency (106). While complete agenesis of the pancreas is incompatible with life, case reports have noted variations in ventral and dorsal agenesis ranging from partial to complete (107, 108).

*HNF1B* mutations have led to the absence of part of the head, body, and tail of the pancreas, suggesting a role for *HNF1B* in dorsal pancreas formation (61–63). Another key regulator, particularly of exocrine function, *PTF1A*, has been implicated in pancreatic and cerebellar agenesis (54). Mutations in downstream enhancers of *PTF1A* have also been noted with isolated pancreatic agenesis (55). Finally, *SOX9* has a role in multiple tissues, and particularly the pancreas through much of its formation. Mutation in one gene results in developmental abnormalities of skeletal, reproductive, and other organs such as pancreas hypoplasia (56, 60).

### Pancreatitis

*SPINK1* is responsible for inhibiting prematurely activated trypsin in the pancreas. Mutations in the gene have resulted in variations in pathology ranging from increased risk of pancreatitis and exocrine pancreatic insufficiency to inconsistent implications in pancreatic disease (109, 110). Overall, it does appear that the gene is implicated in the earlier cause of pancreatitis and has more pancreatic insufficiency than normal cohorts (111). Indeed, case reports have described exocrine pancreatic insufficiency in infants (112).

Cationic trypsinogen (*PRSS1*), anionic trypsinogen (PRSS2), and mesotrypsin (*PRSS3*) are forms of trypsinogen with PRSS1 being the dominant one (104). A hereditary pancreatitis is a rare form of chronic pancreatitis resulting from a mutation in *PRSS1*, which is autosomal dominant with high penetrance and risk of pancreatic adenocarcinoma (113, 114). Episodes of pancreatitis have been noted to be bimodal with peaks around 6 years and 18 years of age, but with variability in pancreatic exocrine function deficiency, though this was often based on clinical symptoms or stool testing (113, 115, 116).

Carboxypeptidases are metalloproteases that play a role in the digestion of proteins and peptides by hydrolyzing C-terminal peptide bonds (117). Following trypsinogen, carboxypeptidase A1 (*CPA1*) is the next most common protein excreted in pancreatic fluid (118). Among a cohort of German individuals, *CPA1* variants were noted to be a risk factor for chronic pancreatitis. Although the mechanism is uncertain, the authors propose misfolding with subsequent stress in the endoplasmic reticulum as a cause (119).

Chymotrypsinogen C variants (*CTRC*) is a calcium-dependent serine protease that is important in cationic trypsinogen activation and trypsin degradation (120, 121). Within the pancreas, however, it appears that it has a role in trypsinogen degradation (121, 122). Therefore, mutations result in loss of function and have been associated with early pancreatitis and chronic pancreatitis in pediatrics (123, 124).

### Maturity onset diabetes of the young (MODY)

Mutations in transcription factors previously noted in the development of the pancreas have been implicated in anatomical variants as well as in endocrine issues, namely, MODY. HNF1B and PDX1 are two that have been implicated (49, 50, 62, 125). Additionally, deficiency in carboxyl ester lipase (CEL) resulted in another form of MODY. CEL, also referred to as bile salt-dependent lipase (BSDL), is one of the four lipases involved in hydrolyses of dietary fat, fat-soluble vitamins, and more specifically cholesterol esters (126). The combined endoscopic pancreatic stimulation test and MRI have shown severely reduced acinar function, along with low pancreas volume with increased lipomatosis in cases of MODY (127, 128).

### Future directions

Knowledge of gene expression and the role of transcription factors allow for further research into stem cell therapy. Current research in both the endocrine and exocrine function was conducted on animal models, though with human pluripotent stem cells (hPSCs). Pluripotent stem cells can be obtained from embryonic cells or can be induced from adult somatic cells, such as fibroblasts or ductal epithelium. Ethical issues are likely to be a big barrier to the use of embryonic cells. Thus, understanding of factors required to convert somatic cells to pluripotent stem cells and then into pancreatic cell lines will hold promise for future. Studies so far seem to show promise in de-differentiating somatic cells-induced pluripotent stem cells (iPSC) and then differentiating into pancreatic progenitor cells (129, 130). In fact, clinical studies are underway in Type 1 diabetes mellitus and the new technologies hold promise in those with exocrine pancreatic dysfunction, particularly in those with chronic pancreatitis requiring islet cell transfer.

There are many answered questions in the human development of the pancreas. How does autonomic innervation, including sympathetic and parasympathetic innervation, develop in the embryo, including when does stimulation such as cephalic input begin? Likewise, a better understanding of innervation by pain fibers may help target therapy.

As current tests for exocrine pancreatic insufficiency include the use of CCK and secretin stimulation, and understanding the maturational process and when the pancreas responds are important.

The use of whole exome sequencing has been increasing with increased access to technology. How certain variants affect exocrine function, and therefore digestion, absorption, and growth will likely provide useful clinical information.

## Conclusion

This review shows a framework for the development of the human pancreas including both gross and microanatomy. Pancreas organogenesis is a stepwise process regulated by a complex network of signaling and transcriptional events, that start with the early endoderm toward pancreatic fate. Many crucial players in this process have been identified, including signaling pathways, genes, regulatory elements, and transcription factors. Much of the work is based on the static evaluation of embryonic and fetal specimens that were available due to ethical issues. While gene expressions and transcription factors involved in pancreas formation have been reported, further understanding of cell-to-cell interaction, including those with stellate cells is necessary. It is possible that with a better understanding of iPSC conversion into various pancreatic cell lineages will help understand better the interaction between these cell types along with gene expression and transcription factor production. Molecular understanding of pancreas formation holds exciting promise for future therapies in both the endocrine and exocrine arms.

## Author contributions

All authors listed have made a substantial, direct, and intellectual contribution to the work and approved it for publication.

## Conflict of interest

The authors declare that the research was conducted in the absence of any commercial or financial relationships that could be construed as a potential conflict of interest.

## Publisher's note

All claims expressed in this article are solely those of the authors and do not necessarily represent those of their affiliated organizations, or those of the publisher, the editors and the reviewers. Any product that may be evaluated in this article, or claim that may be made by its manufacturer, is not guaranteed or endorsed by the publisher.
